# Sequential Onset and Prognostic Impact of Japanese Spotted Fever in a Family Cluster: A Case Report and Brief Literature Review

**DOI:** 10.7759/cureus.111249

**Published:** 2026-06-21

**Authors:** Haruna Nishimaru, Daisuke Miyamori, Naomichi Hirano, Masanori Ito

**Affiliations:** 1 Department of Neurology, Hiroshima City North Medical Center Asa Citizens Hospital, Hiroshima, JPN; 2 Department of General Internal Medicine, Hiroshima University Hospital, Hiroshima, JPN; 3 Department of General Medicine, JA Onomichi General Hospital, Onomichi, JPN

**Keywords:** early diagnosis, eschar, family cluster, japanese spotted fever, tick bite

## Abstract

Japanese spotted fever (JSF) is an increasingly reported tick-borne disease in Japan. Although person-to-person transmission has not been documented, familial clusters have rarely been reported.

We report two cases of JSF in a married couple in their 70s who developed symptoms one week apart after shared weeding activities. The wife presented with the classic triad of fever, rash, and eschar, whereas the husband presented with fever and rash but no identifiable eschar, creating a diagnostic challenge. Following confirmation of JSF in the wife, a skin biopsy specimen and blood samples were obtained from the husband, and the diagnosis was confirmed by polymerase chain reaction.

We reviewed all previously reported familial JSF clusters in Japan and identified 11 patients from five families. Including the present two patients, a total of 13 familial cases were analyzed. Fever was observed in all 13 patients (100%), rash in 12 (92.3%), and eschar in seven (53.8%). The median interval between symptom onset among family members was five days (range = 0-10 days). Among five index cases, four experienced treatment initiation ≥6 days after symptom onset or received no effective JSF-directed therapy, whereas five of six subsequent cases received treatment within five days. Both fatal cases occurred among index patients.

In familial JSF clusters, symptom onset may be staggered despite shared environmental exposure, and eschars may be absent in nearly half of patients. Recognition of an index case may facilitate earlier diagnosis and treatment of subsequently affected family members. Clinicians should consider JSF in patients presenting with unexplained fever and rash, even in the absence of an eschar, and obtain a detailed history regarding family illness, outdoor activities, and potential tick exposure.

## Introduction

Japanese spotted fever (JSF) is a tick-borne rickettsial infection caused by *Rickettsia japonica*, first identified in Japan in 1984 [1]. The disease is transmitted through tick bites and is characterized by the classic triad of fever, erythematous rash, and eschar formation. Although JSF was previously considered endemic mainly to western Japan during the summer season, recent epidemiological studies have demonstrated geographic expansion into eastern regions of Japan and an extended seasonal distribution from spring through winter [2]. Delayed diagnosis remains a clinically important problem because untreated or late-treated JSF may progress to disseminated intravascular coagulation (DIC), multiple organ failure (MOF), and death [3,4].

The clinical diagnosis of JSF can be challenging because its manifestations are often nonspecific during the early phase of illness. Previous studies have reported that eschars are absent in a substantial proportion of patients, with reported frequencies ranging from 44% to 90% [3,5,6]. Consequently, patients presenting with unexplained fever and rash without a clear history of tick bite may initially be misdiagnosed with viral infections or nonspecific febrile illnesses. While the efficacy of combination therapy with tetracycline and levofloxacin has been reported [7], recent nationwide analyses have further demonstrated that delayed initiation of tetracycline therapy significantly increases mortality risk in JSF [8]. Therefore, prompt clinical suspicion and early empirical treatment are essential, particularly in endemic regions.

Familial clusters of JSF are rare because person-to-person transmission has not been documented. However, cohabiting family members may develop the disease sequentially after shared environmental exposure to infected ticks. In such situations, recognition of the index case may facilitate early diagnosis and treatment of subsequent patients, even when atypical findings, such as the absence of eschar, are present. Herein, we report a married couple who developed JSF sequentially after shared exposure during weeding activities. We also review previously reported familial JSF clusters in Japan to clarify the clinical characteristics, diagnostic challenges, treatment delays, and prognostic implications associated with intrafamilial onset.

## Case presentation

**Figure 1:**
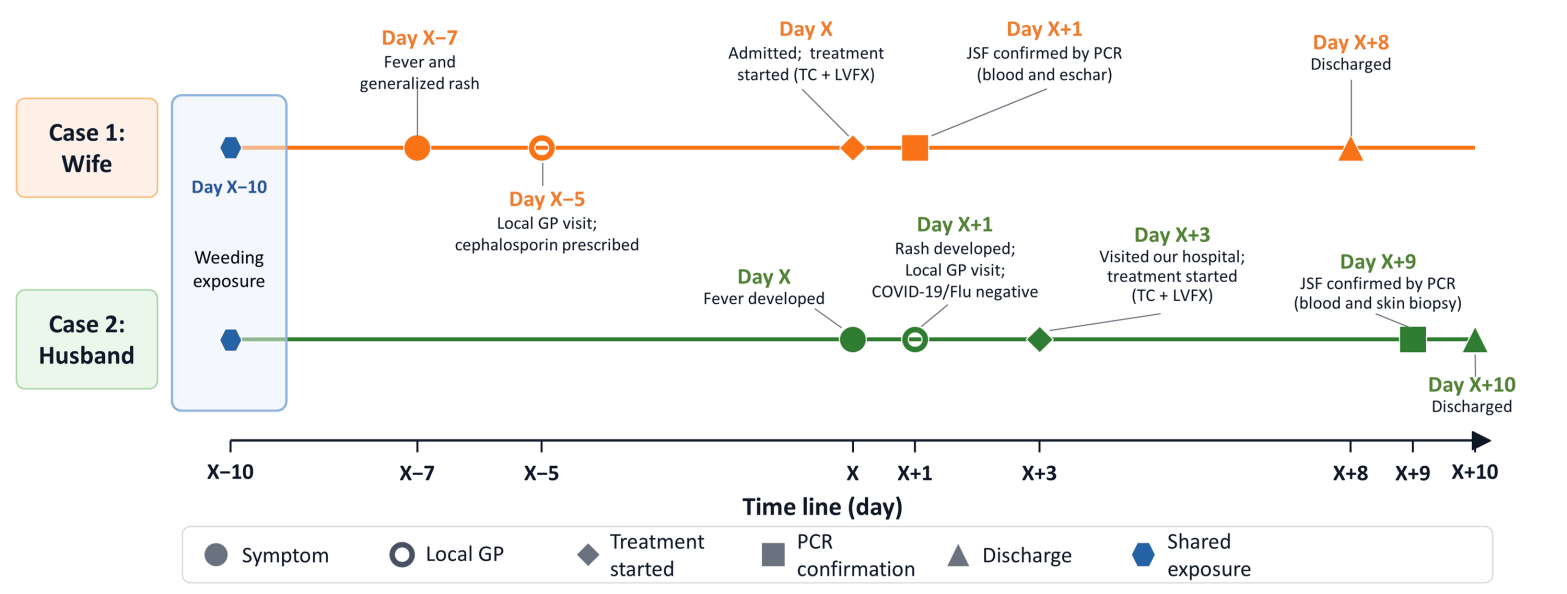
Timeline of onset, diagnosis, and treatment for cases 1 and 2. Day X indicates the day on which case 1 was admitted. The couple had a shared tick-exposure opportunity during weeding on day X-10. Case 1 developed fever and generalized rash on day X-7 and was admitted on day X, when tetracycline and fluoroquinolone therapy were initiated. Case 2 developed fever on day X and rash on day X+1. The confirmed diagnosis in case 1 prompted further evaluation and early treatment of case 2 despite the absence of an eschar. JSF, Japanese spotted fever; PCR, polymerase chain reaction; GP, general practitioner; COVID-19, coronavirus disease 2019; TC, tetracycline; LVFX, levofloxacin. This figure was created using Microsoft PowerPoint (Microsoft Corporation, Redmond, WA, USA).

A married couple in their 70s presented sequentially with febrile illness after shared environmental exposure during weeding around their home in Hiroshima prefecture, Japan. The couple had weeded their garden together approximately 10 days before symptom onset, representing a potential opportunity for tick exposure. The clinical timeline is shown in Figure 1.

**Figure 2:**
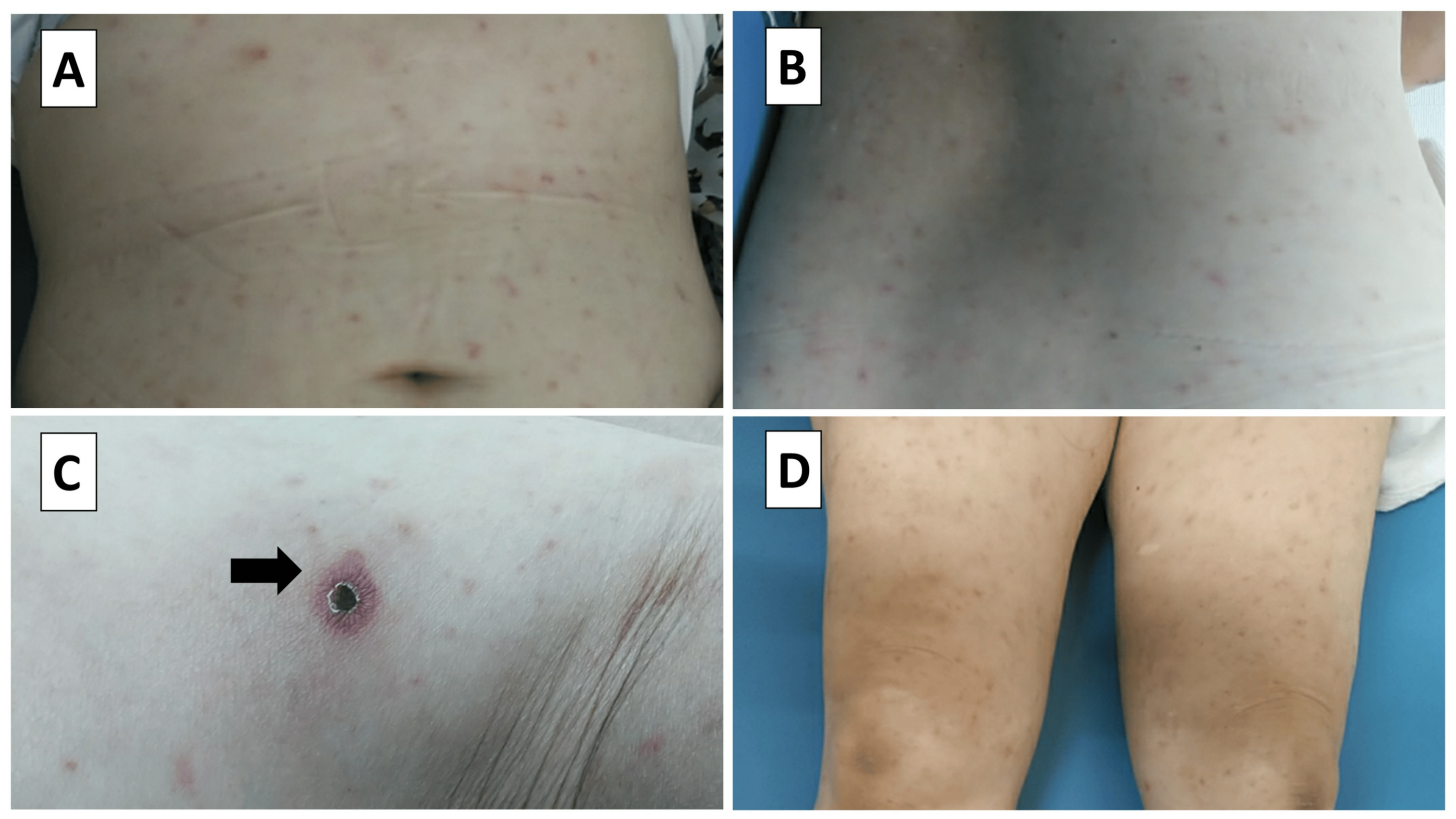
Rashes and eschar in case 1. The images show rashes on the trunk (A), buttocks (B), posterior aspect of the left knee (C), and thigh (D) at the initial consultation for case 1. A biopsy specimen was obtained from the eschar in area C (arrow), and PCR testing confirmed the diagnosis of Japanese spotted fever.

Case 1 was a woman in her 70s with no significant medical history who routinely performed gardening work twice weekly. Seven days before admission, she developed a high-grade fever of approximately 39°C accompanied by a generalized erythematous rash. Five days before admission, she visited a local general practitioner and was prescribed oral cephalosporin antibiotics and antipyretic agents; however, her symptoms progressively worsened. Upon admission to our hospital, her body temperature was 38°C. Physical examination revealed scattered erythematous macules on the trunk and both lower extremities, as well as a black eschar on the posterior aspect of the left knee (Figure 2). Laboratory investigations demonstrated thrombocytopenia, elevated inflammatory markers, and liver dysfunction (Table 1). Based on the classic triad of fever, rash, and eschar, JSF was strongly suspected. Real-time polymerase chain reaction (PCR) assays were performed on blood and eschar specimens at a public health center to detect *Rickettsia japonica,* *Orientia tsutsugamushi* (Kato, Gilliam, Kawasaki, and Kuroki strains), and severe fever with thrombocytopenia syndrome (SFTS) virus. The PCR results were positive for *R. japonica* and confirmed the diagnosis of JSF.

**Table 1:** Laboratory findings on admission for case 1. WBC, white blood cell count; RBC, red blood cell count; Hb, hemoglobin; PLT, platelet count; PT, prothrombin time; PT-INR, prothrombin time-international normalized ratio; APTT, activated partial thromboplastin time; FDP, fibrin/fibrinogen degradation products; TP, total protein; Alb, albumin; AST, aspartate aminotransferase; ALT, alanine aminotransferase; LD, lactate dehydrogenase; ALP, alkaline phosphatase; γ-GT, gamma-glutamyl transferase; T-Bil, total bilirubin; CK, creatine kinase; BUN, blood urea nitrogen; Cre, creatinine; CRP, C-reactive protein; Na, sodium; K, potassium; Cl, calcium.

	Reference range	Case 1		Reference range	Case 1
Hematology			Chemistry		
WBC (/µL)	3,300-8,600	9,160	TP (g/dL)	6.5-8.0	5.8
RBC (10000/µL)	380-500	402	Alb (g/dL)	3.8-5.3	2.7
Hb (g/dL)	11.5-15.0	12.8	AST (U/L)	13-30	279
PLT (10000/µL)	15-35	8.4	ALT (U/L)	7-23	213
Neutrophils (%)	40-70	84.4	LD (U/L)	124-222	558
Lymphocytes (%)	20-45	11.6	ALP (U/L)	38-113	406
Monocytes (%)	2-10	3.8	γ-GT (U/L)	9-32	332
Eosinophils (%)	0-6	0	T-Bil (mg/dL)	0.2-1.2	2.2
Basophils (%)	0-2	0.2	CK (U/L)	41-153	86
Coagulation			BUN (mg/dL)	8-20	18
PT (%)	70-130	100	Cre (mg/dL)	0.46-0.79	0.78
PT-INR	0.9-1.1	1	CRP (mg/dL)	<0.3	17.99
APTT (second)	25-40	37.8	Na (mEq/L)	135-145	138
FDP (µg/mL)	<5	36	K (mEq/L)	3.5-5.0	3.4
			Cl (mEq/L)	98-108	104

On the evening of case 1’s admission, her husband, also in his 70s, developed a fever of approximately 39°C and was designated case 2. His medical history included angina pectoris, hypertension, and diabetes mellitus. The following day, he developed an erythematous rash on the trunk and thighs and visited a local clinic, where influenza and coronavirus disease 2019 (COVID-19) antigen tests were negative. No eschar or obvious tick bite lesion was identified on physical examination, and he was initially diagnosed with a nonspecific febrile illness. However, after his wife’s diagnosis of JSF was confirmed, he presented to our hospital for further evaluation. On admission, he complained of fever, rash, and myalgia. Laboratory findings revealed thrombocytopenia, elevated C-reactive protein (CRP), mild renal dysfunction, and elevated liver enzyme levels (Table 2). Although no eschar was detected, JSF was clinically suspected because of the characteristic rash pattern and shared environmental exposure with the index patient. PCR testing of blood and skin biopsy specimens obtained from the abdominal rash was performed at the public health center for *R. japonica, Orientia tsutsugamushi*, and SFTS virus. The specimens tested positive for *R. japonica* and negative for the other pathogens, confirming the diagnosis of JSF.

**Table 2:** Laboratory findings on admission of case 2. WBC, white blood cell count; RBC, red blood cell count; Hb, hemoglobin; PLT, platelet count; PT, prothrombin time; PT-INR, prothrombin time-international normalized ratio; APTT, activated partial thromboplastin time; FDP, fibrin/fibrinogen degradation products; TP, total protein; Alb, albumin; AST, aspartate aminotransferase; ALT, alanine aminotransferase; LD, lactate dehydrogenase; ALP, alkaline phosphatase; γ-GT, gamma-glutamyl transferase; T-Bil, total bilirubin; CK, creatine kinase; BUN, blood urea nitrogen; Cre, creatinine; CRP, C-reactive protein; Na, sodium; K, potassium; Cl, calcium.

	Reference range	Case 2		Reference range	Case 2
Hematology			Chemistry		
WBC (/µL)	3,300-8,600	10,600	TP (g/dL)	6.5-8.0	7
RBC (10000/µL)	380-500	505	Alb (g/dL)	3.8-5.3	3.8
Hb (g/dL)	11.5-15.0	15.7	AST (U/L)	13-30	61
PLT (10000/µL)	15-35	10	ALT (U/L)	7-23	39
Neutrophils (%)	40-70	90.8	LD (U/L)	124-222	565
Lymphocytes (%)	20-45	4.6	ALP (U/L)	38-113	56
Monocytes (%)	2-10	3.9	γ-GT (U/L)	9-32	20
Eosinophils (%)	0-6	0	T-Bil (mg/dL)	0.2-1.2	1.95
Basophils (%)	0-2	0.8	CK (U/L)	41-153	409
Coagulation			BUN (mg/dL)	8-20	26.5
PT (%)	70-130	76	Cre (mg/dL)	0.46-0.79	1.45
PT-INR	0.9-1.1	1.17	CRP (mg/dL)	<0.3	12.67
APTT (second)	25-40	34.7	Na (mEq/L)	135-145	128
FDP (µg/mL)	<5	13.5	K (mEq/L)	3.5-5.0	4
			Cl (mEq/L)	98-108	95

Both patients were treated empirically with minocycline 400 mg per day for one week and levofloxacin 500 mg per day for five days immediately after hospitalization and before microbiological confirmation. Their clinical conditions improved promptly without progression to DIC or MOF. The wife was discharged eight days after admission, corresponding to 10 days after symptom onset. The husband was discharged seven days after admission, corresponding to eight days after symptom onset.

## Discussion

We reviewed previously reported familial JSF clusters in Japan and identified five prior reports involving 11 patients. Using PubMed, J-STAGE, and Google Scholar as literature databases, we conducted a systematic review with the search terms "Japanese spotted fever" and "family cluster," including their Japanese equivalents (日本紅斑熱 and 家族内発症) when searching Japanese databases. Additionally, a hand search was performed using Google Scholar to ensure comprehensive coverage. The final search was completed on March 1, 2026. The search covered publications from fiscal year 2005 to 2024. Inclusion and exclusion criteria were not explicitly defined in this review. This detailed search strategy enhances reproducibility and thoroughness in identifying pertinent studies.

The search identified five papers (involving a total of 11 individuals) describing cases of intra-family clusters in Japan [4,9-12]. In this study, we reviewed six families, including 13 individuals, comprising five previous cases and a recent case of a married couple (two individuals) from our hospital. Based on the reviewed literature and the current case, a table (Table 3) has been created to outline the variations in clinical features and infection risk among patients from the same family. The extracted items were age and sex, fever, erythema, eschars, and their location.

**Table 3:** Clinical characteristics and exposure history in familial clusters of Japanese spotted fever.

Reference (Year, Region)	Patient	Fever	Rash/erythema	Eschar	Environmental exposure
Present case, Hiroshima	Wife (73, Female)	Yes (38°C)	Trunk and lower extremities	Yes (posterior left knee)	Shared weeding
	Husband (76, Male)	Yes (39°C)	Abdomen and thighs	No	Shared weeding
Kobayashi et al. (2022), Chiba [9]	Husband (87, Male)	Yes (40.3°C)	Trunk, extremities, palms, soles	No	Forestry work
	Wife (78, Female)	Yes (39.5°C)	Trunk, extremities, palms, soles	Yes (right earlobe)	Suspected indoor exposure
Matsuura et al. (2018), Kagawa [4]	Husband (80, Male)	Yes (39.6°C)	Trunk and extremities	Yes (left popliteal fossa)	Hobby farming
	Wife (74, Female)	Yes (39.6°C)	Generalized rash	Yes (left lower leg)	Hobby farming
Hashimoto et al. (2016), Saga [10]	Daughter (46, Female)	Yes (38°C)	Trunk and extremities	Yes (right axilla)	Mountain cleaning
	Father (78, Male)	Yes	Trunk and palms	No	Mountain cleaning
Nishiguchi et al. (2019), Wakayama [11]	Mother-in-law (94, Female)	Yes	No rash	No	Suspected indoor exposure via dog
	Wife (65, Female)	Yes (38°C)	Lower abdomen and thighs	No	Suspected indoor exposure via dog
	Husband (68, Male)	Yes (38°C)	Trunk, extremities, palms, soles	No	Weeding around the house
Kubozono et al. (2006), Kagoshima [12]	Husband (65, Male)	Yes (39.0°C)	Almost the entire body	Yes (right thigh)	Camping
	Wife (59, Female)	Yes (39.2°C)	Almost the entire body	Yes (left thigh)	Camping

The median age was 73, and the male-to-female ratio was 6:7. Although most patients had engaged in outdoor activities where exposure to ticks was possible, in two families, infection via ticks brought into the home or via pets was suspected. Fever was observed in all the patients (13/13, 100%). Erythema was the next most common symptom, observed in 12 patients (92.3%); however, eschars were confirmed in only seven patients (53.8%).

We also extracted environmental exposure, order of onset, interval between family members’ symptom onset, time from symptom onset to treatment, and outcome, as shown in Table 4.

**Table 4:** Onset timing, treatment delay, and outcomes in familial clusters of Japanese spotted fever.

Reference	Patient	Order of onset	Interval within family	Time from symptom onset to treatment	Outcome
Present case	Wife	Index case	—	7 days	Recovered
	Husband	Second case	7 days later	3 days	Recovered
Kobayashi et al. [9]	Husband	Index case	—	8 days	Recovered
	Wife	Second case	4 days later	4 days	Recovered
Matsuura et al. [4]	Husband	Index case	—	No effective treatment	Died
	Wife	Second case	Few days later	4 days	Recovered
Hashimoto et al. [10]	Daughter	Index case	—	1-2 days	Recovered
	Father	Second case	5 days later	0-1 days	Recovered
Nishiguchi et al. [11]	Mother-in-law	Index case	—	No effective treatment	Died
	Wife	Second case	6 days later	8 days	Recovered
	Husband	Third case	10 days after wife’s onset	4 days	Recovered
Kubozono et al. [12]	Husband	Simultaneous onset	Same day	5 days	Recovered
	Wife	Simultaneous onset	Same day	5 days	Recovered

Excluding the family reported by Kubozono et al. [12], who developed symptoms on the same day, the median interval between the index case and the second case was six days, with a range of four to 10 days. Furthermore, excluding the aforementioned family, among the index cases within a family, four out of five cases required six days or more from symptom onset to treatment or remained untreated. In contrast, among the second and subsequent cases within the same family, five out of six cases were treated within five days. Fatal cases occurred among index patients.

Previous studies have suggested that delayed initiation of tetracycline therapy beyond six days after symptom onset is associated with an increased risk of severe disease and mortality in JSF [8]. However, the number of familial cases available for analysis remains small, and our findings do not establish a causal relationship between recognition of an index case and improved clinical outcomes. Rather, they suggest that awareness of a preceding family member with JSF may facilitate earlier clinical suspicion and more timely initiation of appropriate antimicrobial therapy in subsequently affected individuals.

The present husband-and-wife cases illustrate this point. Although the husband lacked an identifiable eschar and was initially considered to have a non-specific febrile illness, the diagnosis of JSF in his wife prompted reconsideration of the diagnosis and early initiation of effective treatment. Therefore, when evaluating patients with unexplained fever and rash, clinicians should obtain a detailed history regarding recent illness among family members, shared outdoor activities, and potential tick exposure, even when characteristic eschars are absent.

The reviewed cases were familial clusters with an epidemiological link through shared exposure or prior onset in another family member. This link may have facilitated diagnosis in subsequent cases compared with sporadic cases. In these cases, the detection rate of eschars was approximately 50%. This result cautiously suggests the possibility that some atypical JSF cases, which lack eschars or a clear history of tick bites, might be misclassified as nonspecific febrile illnesses in clinical practice. Consequently, the true incidence of JSF could be underestimated compared to current reports. Further investigation with larger datasets is needed to clarify this potential under-recognition.

Accurate diagnosis of atypical initial cases is essential to save lives and to enable early detection and treatment of family members exposed to the same environment who may develop the disease during the incubation period. Despite common exposure, the onset of symptoms may occur at different times, and a significant number of patients may not exhibit eschars. It is crucial to obtain a comprehensive history of illnesses among family members, their outdoor activities, as well as potential pet-related and indoor tick exposures.

## Conclusions

This case report and literature review highlight differences in treatment timing and outcomes between index and subsequent cases in familial JSF clusters. Despite common environmental exposure, the onset of symptoms among family members can differ, with eschars frequently absent. Early identification of JSF, particularly in index cases, is challenging yet crucial, as delayed treatment is associated with poor outcomes, including fatalities. A comprehensive family history and awareness of outdoor or pet-related exposure are vital for prompt diagnosis and antibiotic intervention. While these findings highlight the possible benefits of prompt recognition and treatment in familial JSF clusters, further studies with larger sample sizes are needed to confirm these observations and clarify their impact on morbidity and mortality.
